# Sulphuric Acid and Peroxide of Sodium in the Treatment of Pulpless Teeth

**Published:** 1895-02

**Authors:** F. T. Van Woert

**Affiliations:** Brooklyn, N. Y.


### SULPHURIC ACID AND PEROXIDE OF SODIUM IN THE TREATMENT OF PULPLESS TEETH.

BY F. T. VAN WOERT, M.D.S., BROOKLYN, N. Y.

THE object of this short paper is to explain somewhat the details necessary for the accomplishment of results such as I have attributed to the above named drugs; and in the beginning be it understood that there is nothing to follow which is original with me. The credit, if any, is due to the gentlemen whose names appear in this paper as having introduced these remedies to the profession. Since the introduction of sodium peroxide by Dr. E. C. Kirk, three years ago, I have met with very great success in its use, as well as in making the solution. I seem to have been more fortunate than many others, as I am constantly in receipt of communications stating that the writers have failed utterly in their efforts to accomplish results like those claimed for the remedy. The sulphuric acid which was recommended by Dr. Callahan at Asbury Park, last August, I have found so valuable in bringing to light nerve-canals that would never be found were it not for its use, that I embrace this opportunity to spread the glad tidings, with the hope that others may be profited by it as I have. It is generally conceded that one of the most difficult and uncertain operations which we are called upon to perform is that of opening and sterilizing roots, and in a great many cases it is utterly impossible to accomplish that end, and the result is the loss of many valuable teeth. Dr. Shields, of New York, claims that this is due to a lack of professional education and manipulation, and makes the absurd statement that he always opens to the end and fills all roots, which you know as well as I do is a mechanical impossibility. It is eighteen hundred and ninety-four years since any one man has claimed such perfection, and I predict it will be as many more before another member of our profession will have the audacity to proclaim himself absolutely perfect, and the rest of us diabolical quacks. My excuse for these utterances will be found on pages 12 to 15 in the *Transactions* of the New Jersey State Dental Society, 1893.

Dr. Callahan recommends a forty- to fifty-per-cent. aqueous solution, as follows: "Let us suppose we have an inferior molar tooth in which the pulp has been destroyed; we adjust the rubber dam, open the pulp-chamber thoroughly, take an old discarded broach, twist a little cotton on the end, bend the broach to a right angle so it will reach well down into the cavity, place the broach into a suitable handle, and by means of the broach and cotton place directly upon and above the dead pulp a drop or two of a forty- to fifty-per-cent. aqueous solution of sulphuric acid. The solution, by a process of dehydration, will cause the pulp to shrink and toughen so that it can with comparative ease be removed. Now, by means of the broach and cotton, place a drop of the solution over the entrance of each canal. Sometimes it will be necessary to sink a little well or depression at the mouth of the canal, to get the acid to stay where it is wanted, being careful to use only round or bud-drills for this purpose.

"Take a No. 5 Donaldson nerve-canal cleaner, bend it to a suitable angle, cut the shank short with nippers so the broach will fit up close to the handle and be rigid and strong; then with a pumping motion begin to enter the canal slowly and carefully. The acid will precede or follow closely the fine broach and destroy all septic matter it comes in contact with. Proceed until the patient notifies you of a sensation which is similar to that felt when chlora-percha goes through the foramen; treat all of the canals in the same manner. I say all, because sometimes you will find what appears to be four distinct canals.

"Usually three canals will be found. The posterior root will have one broad canal; the anterior root will nearly always show what seems to be two canals.

"By this time the solution will be so charged with disintegrated tooth- and pulp-substance that it will hide the canals from view. Now, by means of a Dunn syringe, fill the cavity with a saturated solution of bicarbonate of soda; this, when brought in contact with the acid solution, liberates carbonic acid gas in such quantities that the effervescence will carry all the broken-up tooth- and pulp-substance out of th ecanal, out of the tooth onto the rubber dam, leaving a deposit of bicarbonate of soda lining the whole tooth. This can be removed, if desired, by a little sterilized water, alcohol, or peroxide, either of which will leave the canals white and clean.

"If we desire to make the canals larger, place more acid in them and use a larger broach until the canal is as large as wanted; then cleanse again with bicarbonate of soda; dry the canals thoroughly by means of paper points, alcohol, hot air, etc., and you have the cavity and all the canals thoroughly opened, thoroughly clean, thoroughly aseptic, and you can proceed to treat or fill, as you may choose."

I have been using this preparation as described in the above quotation, and find the claims made by Dr. Callahan for it precisely as he has stated, to wit,—

1. The operation is perfectly safe, because the action of the acid is self-limiting on dentine.
2. It is a pronounced germicide.
3. The acid acts upon diseased tissue with far greater vigor than healthy.
4. The destroying of the diseased tissue in this way leaves a fresh aseptic surface.
5. An aseptic wound will heal itself in any part of the body if properly closed.
6. Dr. Callahan claims that the acid softens the dentine a very short distance.

In the use of a bicarbonate of soda solution the acid is neutralized, in doing which carbonic acid gas is generated in sufficient quantities to carry off the *débris* from the root-canals.

Now, do not understand either Dr. Callahan or myself as claiming the use of sulphuric acid and soda bicarbonate solutions to make it possible to open all root-canals, but credit us with the conviction that by its use many hopeless cases are mastered and hundreds of teeth saved which would otherwise be lost.

When you are not successful in your attempts to thoroughly cleanse the canals, place in the pulp-chamber a saturated solution of sodium peroxide and seal the crown from twenty-four to forty-eight hours; then remove and wash with warm water, after which fill in the usual manner, with the assurance that the majority of cases will not give you further trouble. But to obtain these results it is absolutely necessary that every detail is followed in the making of the solution.

First, the peroxide must be powdered in a mortar, as it is not fine enough as purchased to add to the water without a chance of spoiling the solution before its completion.

Dr. Kirk explains the cause of this on page 499 of the *Dental Cosmos*, June, 1894, as follows: "If the solution be made hurriedly by the addition of considerable quantities of the powder to the water at one time, the evolution of heat due to the energy which attends the combination produces a rapid elevation of the temperature of the solution.

"This causes a decomposition of the peroxide, a loss of its loosely combined extra atom of oxygen occurs, and the resulting solution is little more than a solution of sodium hydrate, or ordinary caustic soda, which is practically inert as far as bleaching power is concerned.

"To obviate the rise of temperature and consequent decomposition of the peroxide, the solution must be made slowly."

To make this solution, take a common tumbler about half full of distilled water, place it in the centre of a good-sized pudding-dish, and pour all the cold water around it possible without floating the glass. Add the sodium peroxide in very small portions, about what could be taken upon the point of the large blade of a pocket-knife, dusting it in the water slowly to cause as little agitation as possible, and this amount should not be added oftener than once in a half-hour, being careful to have the sodium peroxide finely powdered; this to be continued until the preparation begins to look opaque as powder is added. Let it stand over night, and it is then ready for use. This takes several days to make, but it will more than pay for the time consumed in its prompt action as a bleacher and sterilizer.

The general impression is that sodium peroxide is for bleaching only, while it is the most valuable preparation I have ever found for the treatment of dead teeth, if used as described before.

The question has been raised, as to whether this solution does not disintegrate tooth-substance. I feel safe in saying that it does not, having used it very extensively the last three years without once giving trouble. The preparation can be kept in a glass-stoppered bottle for a long time. It is just as well to keep it in a cool place.

To preserve the powder, screw the top of the can down tight, and run between it and the outer rim a little base wax melted so that it will barely pour. You can remove the top at any time by simply passing the point of a knife through the wax, close to and around the same.

I sincerely hope I have made myself plain in this matter, and that those who read this article will try for the results claimed, as I am sure they will find the addition to their medicament cases very valuable. I am just in receipt of a communication which reports very successful results in the use of peroxide of sodium by a student in the Philadelphia College, after the following method: Apply the dam, cleanse and prepare the cavity, place in the pulp-chamber a small portion of the powdered peroxide, then flood the cavity with water, allowing it to remain until the agitation from the combination ceases, after which it is washed out and treated as before described. This would seem to me a very practical and sure way of obtaining a solution that would be effectual.

